# Genetic Heterogeneity Underlying Phenotypes with Early-Onset Cerebellar Atrophy

**DOI:** 10.3390/ijms242216400

**Published:** 2023-11-16

**Authors:** Dolores Martínez-Rubio, Isabel Hinarejos, Herminia Argente-Escrig, Clara Marco-Marín, María Ana Lozano, Nerea Gorría-Redondo, Vincenzo Lupo, Itxaso Martí-Carrera, Concepción Miranda, María Vázquez-López, Asunción García-Pérez, Ana Victoria Marco-Hernández, Miguel Tomás-Vila, Sergio Aguilera-Albesa, Carmen Espinós

**Affiliations:** 1 Rare Neurodegenerative Diseases Laboratory, Valencia Biomedical Research Foundation, Centro de Investigación Príncipe Felipe (CIPF), 46012 València, Spain; 2 Joint Unit CIPF-IIS La Fe Rare Diseases, 46012 València, Spain; 3 Department of Neurology, Hospital Universitari Arnau de Vilanova, 46012 València, Spain; 4 Structural Enzymopathology Unit, Instituto de Biomedicina de Valencia (IBV), Consejo Superior de Investigaciones Científicas (CSIC), 46022 València, Spain; 5 Centro de Investigación Biomédica en Red de Enfermedades Raras (CIBERER), Instituto de Salud Carlos III (ISCIII), 28220 Madrid, Spain; 6 Paediatric Neurology Unit, Department of Paediatrics, Hospital Universitario de Navarra, Navarrabiomed, 31008 Pamplona, Spain; 7 Paediatric Neurology Unit, Department of Paediatrics, Hospital Universitario Donostia, 20014 Donostia, Spain; 8 Paediatric Neurology Unit, Department of Paediatrics, Hospital General Universitario Gregorio Marañón, 28027 Madrid, Spain; 9 Paediatric Neurology Unit, Department of Paediatrics, Hospital Universitario Fundación Alcorcón, Alcorcón, 28922 Madrid, Spain; 10 Paediatric Neurology Unit, Department of Paediatrics, Hospital Universitari Doctor, Peset, 46017 València, Spain; 11 Paediatric Neurology Unit, Department of Paediatrics, Hospital Universitari i Politècnic La Fe, 46026 València, Spain; 12 Biotechnology Department, Universitat Politècnica de València, 46022 València, Spain

**Keywords:** ataxia, cerebellar atrophy, rare disease, neuroimaging, exome sequencing, gene panel

## Abstract

Cerebellar atrophy (CA) is a frequent neuroimaging finding in paediatric neurology, usually associated with cerebellar ataxia. The list of genes involved in hereditary forms of CA is continuously growing and reveals its genetic complexity. We investigated ten cases with early-onset cerebellar involvement with and without ataxia by exome sequencing or by a targeted panel with 363 genes involved in ataxia or spastic paraplegia. Novel variants were investigated by *in silico* or experimental approaches. Seven probands carry causative variants in well-known genes associated with CA or cerebellar hypoplasia: *SETX, CACNA1G, CACNA1A, CLN6*, *CPLANE1*, and *TBCD*. The remaining three cases deserve special attention; they harbour variants in *MAST1, PI4KA* and *CLK2* genes. *MAST1* is responsible for an ultrarare condition characterised by global developmental delay and cognitive decline; our index case added ataxia to the list of concomitant associated symptoms. *PIK4A* is mainly related to hypomyelinating leukodystrophy; our proband presented with pure spastic paraplegia and normal intellectual capacity. Finally, in a patient who suffers from mild ataxia with oculomotor apraxia, the *de novo* novel *CLK2* c.1120T>C variant was found. The protein expression of the mutated protein was reduced, which may indicate instability that would affect its kinase activity.

## 1. Introduction

The classic role of the cerebellum is to be an efficient modulator of movements. Cerebellar dysfunction is traditionally related to cerebellar motor syndrome, which is characterised by impairments of gait, limb coordination, speech, and ocular movement control [1]. Along with the motor impairment, early damage to the cerebellum leads to cognitive, behavioural and emotional consequences, as the developing nervous system appears to require an intact cerebellum for normal cognitive development [2].

Early-onset ataxias usually present with a relevant disability, including imbalance, poor coordination, developmental delay, and intellectual disability (ID) [3,4]. Cerebellar atrophy (CA) is a key neuroimaging finding usually associated with paediatric forms of cerebellar ataxias of postnatal-onset metabolic or genetic origin [5]. CA identification requires serial neuroimaging studies, as in one it cannot be distinguished from cerebellar hypoplasia, most associated with non-progressive neurodevelopmental disorders [6,7,8]. However, the number of disorders that present with CA is ever-increasing with an extremely broad clinical spectrum, including rare neurodegenerative disorders and developmental disorders [7,9,10,11,12]. Due to rapid advances in technology based on NGS (Next Generation Sequencing), genetic analyses are included in clinical practice and have made possible the discovery of hundreds of genes involved in CA. In the paediatric age group, most cases are due to autosomal recessive (AR) conditions, and a high frequency of cases caused by *de novo* disease-causing mutations transmitted in an autosomal dominant (AD) manner is appreciated [4]. Its diagnosis is a challenge. The search for cerebellar atrophy (HP:0001272) at HPO (Human Phenotype Ontology; https://hpo.jax.org/app; accessed on 14 September 2023), yields associations with 579 genes.

Among the cerebellar ataxias, we find ARCAs (Autosomal Recessive Cerebellar Ataxias) or SCARs (AR Spinocerebellar Ataxias), and ADCAs (Autosomal Dominant Recessive Cerebellar Ataxias) or SCAs (AD Spinocerebellar Ataxias), in which ataxia is a prominent clinical feature or is a sign included in the clinical phenotype [12,13,14]. This classification provides insufficient guidance for physicians and researchers, so alternatives for recessive cerebellar ataxias have been generated that could be more effective, including the gene name and based on a division between phenotypes with ataxia (e.g., ATX-FXN, Friedreich ataxia), ataxia with a predominant movement disorder (e.g., ATX/HSP-HEXA, Tay-Sachs disease), or complex phenotypes that occasionally present with CA (e.g., *EXOSC3*, pontocerebellar hypoplasia type 1B) [15]. The list of clinical forms, available on the web page of the International Parkinson and Movement Disorder Society (Taskforce on nomenclature in movement disorders), is continually growing as new genes are described.

We describe here ten probands who show CA with or without ataxia, in which a brain magnetic resonance imaging (MRI) was performed due to a motor impairment or early signs of a neurological disease. These patients were selected from a national cohort of children with clinical signs of an early-onset movement disorder and/or ataxia, and CA identified by brain MRI. Causative genes are involved in ataxia phenotypes but are also associated with channelopathies, neuronal ceroid lipofuscinosis, Joubert syndrome (JS) and encephalopathies, which highlights the wide spectrum of genotypes associated with CA. In this cohort, we found relatively frequent genes, *SETX, CACNA1G, CACNA1A, CLN6*, *CPLANE1*, and *TBCD*, with well-known-disorders, new clinical phenotypes related to not-so-common genes, *MAST1* and *PI4KA*, and a novel candidate gene, *CLK2*, which may act as causative gene or contribute to the clinical phenotype. Additionally, *in silico* or experimental studies were performed to ascertain the pathogenicity of novel variants, which has been demonstrated essential to achieve conclusive results [7,8,9,16,17,18]. 

## 2. Results

**Figure 1:**
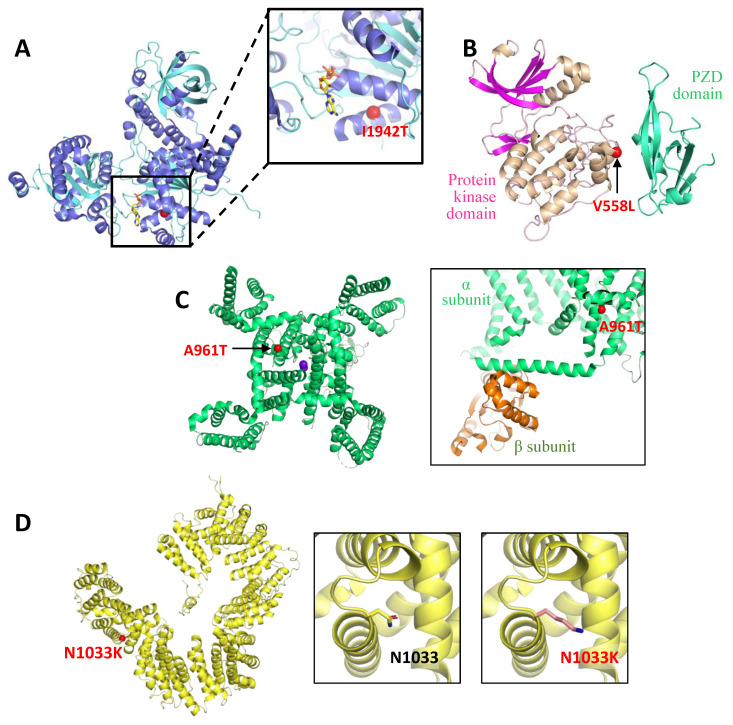
Structural modelling. (**A**) The crystallographic structure of the yeast SEN1 helicase domain (PDB entry 5mzn) is shown in the cartoon representation. Residue corresponding to I1942 in human senataxin (SETX), where missense mutation p.I1942T has been identified, is marked with a red sphere, to show its proximity to de nucleotide binding site. The inset shows a close-up view of the region where the mutation is located. The nucleotide is shown in stick representation with carbon, oxygen, nitrogen, and phosphorus atoms coloured yellow, red, blue, and orange respectively. (**B**) Cartoon representation of the protein kinase and pzd domains of the model generated by AlphaFold for human MAST1 (accession number Q9Y2H9). Localization of p.V558L is represented with a red sphere. (**C**) Cartoon representation of the cryo-electron microscopy structure for the calcium translocating pore of human Ca_v_3.1 (PDB entry 6kzo), from the cytosolic region. Ca^2+^ are shown as purple spheres. The red sphere localizes residue A961, where p.A961T has been identified. The inset shows a close-up of the region where this mutation is localized, in the structure of the voltage-gated calcium channel Cav1.1 from rabbit (PDB entry 5gjv), to show the proximity of the mutation to the elements from the α subunit that mediate the interaction with the β regulatory subunit of this channel. (**D**) Cartoon representation of the alpha solenoid structure predicted for TBCD (AlphaFold model; accession code Q9BTW9). Localization of p.N1033K is mapped with a red sphere. The inset shows in detail the region where N1033 is located, with its side chain representing a stick, and on the right, the structural model of the p.N1033K mutation.

The patient MD-359 (*SETX*, Table 1 and Table 2), was an 8 year-old female who was referred at 6 years of age because of severe learning difficulty at school. She came from a consanguineous family. She had four other siblings, two of whom suffered from ID with no ataxia or CA signs in the MRI. Her IQ (Intelligence Quotient) was within normal limits (WISC-IV, Wechsler Intelligence Scales for Children Fourth Edition, total IQ: 92), but she still required support at school and her academic performance remained poor. She also suffered from bilateral sensorineural deafness, and gait ataxia was manifested at the exam with no other cerebellar signs or dysmorphias. Height and weight were normal for their age. Complete blood tests including lactic acid, liver, kidney, hormones, and metabolic panel were normal except for mildly increased α-fetoprotein (13.6 µg/L, normal range 0.9−8.8). Nerve conduction velocities at 12 years of age were normal and two brain MRIs performed at 8 and 11 years old revealed a mild to moderate CA. By WES (Whole Exome Sequencing)-proband, the c.5825T>C (p.I1942T) variant was identified in homozygosis in *SETX*, which has been widely associated with SCAR previously (Table 1). The variant *SETX* c.5825T>C was screened in her parents and siblings by Sanger sequencing and all of them carried the mentioned mutation in heterozygosis, except a sister who did not have the mutation. For senataxin (SETX), there is no experimental structure, and the model generated by AlphaFold displays a predominant absence of secondary structure. However, the folding for the C-terminal region of senataxin, where the missense clinical mutation p.I1942T is located, it has been predicted to contain a helicase domain with homology to human RENT1, the immunoglobulin μ-binding protein 2 (IGHMBP2), and yeast-splicing endonuclease 1 (SEN1) [19]. Based on the senataxin model generated by AlphaFold and the structure of yeast SEN1, it can be inferred that the mutation p.I1942T located in the RecA1 domain of the helicase folding (Figure 1A), is distant from the RNA-binding groove but in proximity to the nucleotide-binding site, although not directly involved in nucleotide binding. The side chain of I1942 is oriented towards the core of the RecA1 domain interacting with a conserved helix. Therefore, it is likely that this mutation, which changes a hydrophobic residue to a polar one, destabilises the folding of this domain, altering its stability.

**Figure 2:**
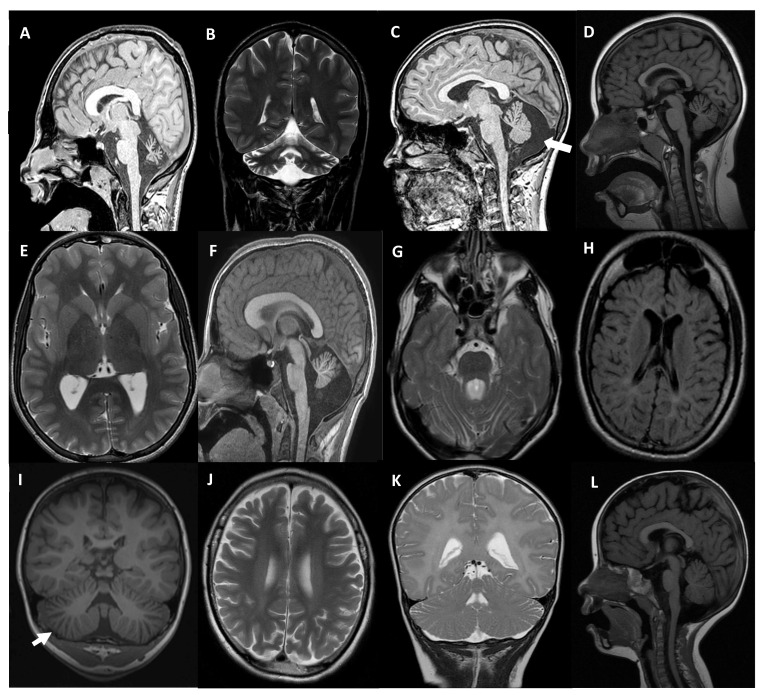
Main neuroimaging signs. (**A**,**B**) MD-353/*CACNA1G*, severe vermian atrophy in mid-sagittal T1-weighted image (**A**) and mild global atrophy in both cerebellar hemispheres in coronal T2 (**B**) at 17 years of age. (**C**) MD-309/*CACNA1A*, a 12 year-old with moderate atrophy in the anterior lobe of the cerebellar vermis and a mega cisterna magna in mid-sagittal T1 (white arrow). (**D**) MD-556/*CACNA1A* at 10 years, moderate atrophy in the anterior lobe of the cerebellar vermis (T1-weighted sagittal image). (**E**,**F**) MD-548/*MAST1* at 13 years, T2 axial image showing thinning of anterior arm of internal capsule (**E**), and T1 mid-sagittal image with a globally increased corpus callosum and vermian reduced volume (**F**). (**G**) MD-392/*CPLANE1* at 17 years, T2 axial image showing a lack of normal decussation of superior cerebellar peduncular fiber tracts that follow and horizontal course (molar tooth sign). (**H**) MD-297/*TBCD* at 18 years, T2 FLAIR axial image revealing a striking reduction of supratentorial white matter, severe brainstem and cerebellar atrophy was also ascertained. (**I**,**J**) MD-471/*CLN6*, at 5 years (**I**), T1 coronal image showing enlarged cerebellar folia spaces (white arrow) at 6.5 years (**J**), T2 axial image disclosed cortical atrophy and deep/periventricular white matter hyperintensities. (**K**) MD-610/*PI4KA*, at 30 months, T2 coronal image showing diffuse hypomyelination and enlarged cerebellar folia interspaces. (**L**) MD-436/*CLK2* at 20 months of age, with mild enlargement of folia spaces in the anterior lobe of the cerebellar vermis in mid-sagittal T1.

The patient MD-353 (*CACNA1G*, Table 1 and Table 2), was a 21 year-old who was referred at the age of 11 and presented with arthrogryposis and severe global developmental delay (sitting at 3 years old, not able to walk independently). Regarding his familiar history, a 6 years old male first-degree cousin had spastic paraplegia, a second-degree male cousin had ID, and another second-degree male cousin suffered from autism. He attended a special needs school, and his expressive language was very limited, although his comprehension was preserved. He used a wheelchair and ankle-foot orthoses and needed help with basic daily activities. At the exam, he had horizontal and vertical spontaneous nystagmus, severe contractures in upper limbs with hypoplastic claw hands, reduced muscular tone in the lower limbs and absence of deep tendon reflexes. A brain MRI showed CA, evident from 3 years of age (Figure 2A,B). The *CACNA1G* c.2881G>A (p.A961T) variant was observed in the patient in a heterozygous state; his parents did not carry the variant. *CACNA1G* c.2881G>A has been previously reported (Table 1). CACNA1G is a low voltage-gated Ca^2+^ channel, also known as Cav3.1, that mediates the entry of Ca^2+^ into excitable cells [21]. The residue A961 is located in the α subunit of this protein complex, particularly in one of the α helices that constitute the core of the Ca^2+^ translocating pore (Figure 1B). However, the side chain of A961 is not oriented towards the pore but towards the cytoplasmic region of the channel; the change to threonine is not expected to affect the folding of the protein. Insights from the structure of the rabbit Cav1.1 channel [22] suggest that the p.A961T mutation could potentially affect the intracellular segment required for binding to the β regulatory subunit of this channel (Figure 1B); both elements are not visible in the human Cav3.1 structure. This interaction could be crucial for the proper functioning of the channel.

The patient MD-309 (*CACNA1A*, Table 1 and Table 2) was a 12 year-old male who was referred when he was 4. He was born to non-consanguineous healthy parents and presented with a mild global developmental delay in language acquisition, mild intellectual disability (WISC-IV total IQ: 61 at 4 years of age), and a 1 h duration of disabling episodes of instability since aged 2 (sometimes triggered by fever). At 4 years old, he had a severe spontaneous horizontal rotatory nystagmus, absence of deep tendon reflexes, upper limb dysmetria, and gait ataxia without dysarthria or tremor. Complete blood tests including lactic acid, liver, kidney, hormones, α-fetoprotein, and metabolic panel were normal. Nerve conduction velocities and somatosensory evoked potentials were also normal, but two brain MRIs performed three years apart showed a moderate atrophy in the anterior lobe of the cerebellar vermis and a mega cisterna magna (Figure 2C). Using the gene panel SPGAtaxia-363, the novel *CACNA1A* c.3042C>G (p.Y1014*) change was identified in the patient. Further segregation analysis showed that this mutation was *de novo*.

The patient MD-556 (*CACNA1A*, Table 1 and Table 2), a 14 year-old female, presented from 2 months to 12 months of age with sudden episodes of pallor, sweating, hypotonia, stillness, ocular horizontal abnormal movements, and vomiting. They occurred monthly and lasted from minutes to several hours. A neurological examination was normal between episodes except intermittent strabismus. Investigations were unremarkable, including brain MRI, electroencephalogram (EEG), and serial extended blood and urine analysis. Psychomotor development was normal afterwards but at school age she was diagnosed with attention deficit and learning disorder with a WISC-IV total IQ of 91. Methylphenidate improved attention symptoms and school performance. At 12 years, her learning performance was declining, and an updated total IQ was 73. From 13 years, she presented with vertigo-like episodes with gait instability. A control brain MRI revealed a mild vermian atrophy (Figure 2D). An EEG showed intermittent bihemispheric spike and wave complexes without ictal phenomena. Acetazolamide did not improve symptoms, but topiramate did. WES-trio revealed the *de novo* deletion of c.234_235delCT (p.F79Pfs*22) in *CACNA1A*, was, to date, not associated with disease in heterozygosis in the patient, predicted as pathological according to the ACMG (American College of Medical Genetics and Genomics)/AMP (Association for Molecular Pathology) criteria [20] (Table 1). 

MD-548 (*MAST1*, Table 1 and Table 2) was the only son of non-consanguineous healthy parents, with an unremarkable family history. During pregnancy, foetal movements were decreased, but no other signs were noticed and delivery was normal. From the first months of life, psychomotor retardation was detected. At 8 months of life, he presented with regression of some developmental milestones with a decreased contact with the environment and less activity. An array-CGH (Comparative Genomic Hybridization) analysis revealed no findings of interest. A brain MRI performed at 6 months showed a small cerebellar vermis with enlarged folia interspaces. At the age of 12, vermis folia interspaces were mildly enlarged but remarkably, it was disclosed a bilateral thinning of the anterior arm of the internal capsule resembling a pseudo-fusion of putamen and caudate nuclei, and an increase in the overall size of the corpus callosum (Figure 2E,F). Currently, the patient is 14 years old, he presents an absence of verbal language, severe ID, hypotonia and truncal ataxia, but he is able to walk autonomously. The genetic analysis by WES-trio detected a *de novo* novel variant in *MAST1*, c.1672G>C (p.V558L) in heterozygosis, predicted as damaging according to the ACMG/AMP criteria (Table 1). 

**Figure 3:**
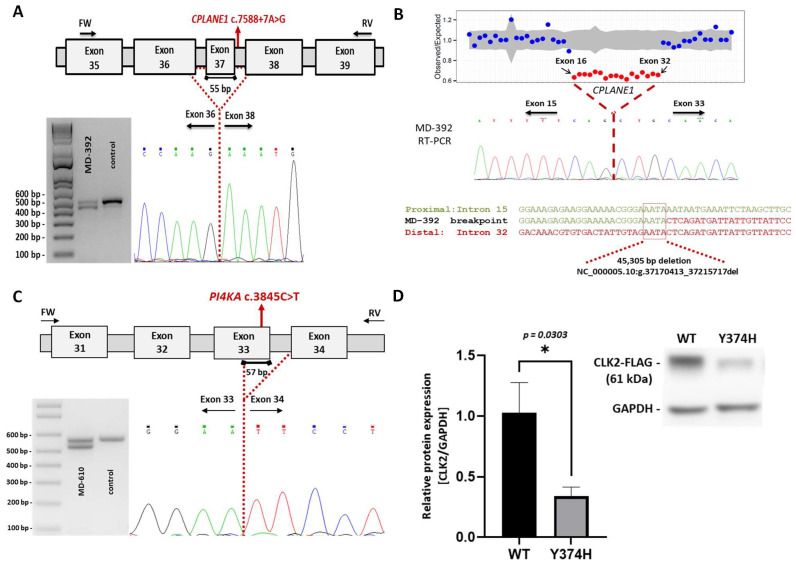
Studies to investigate the pathogenicity of variants of interest. (**A**) Transcript analysis of *CPLANE1* c.7588+7A>G. Schematic representation of the aberrant splicing. Exons are indicated by boxes and introns are represented by horizontal bars (not to scale). Location of specific primers used in RT-qPCR are indicated by arrows. Dotted lines represent the anomalous splicing outcome and sequence of the abnormal product is shown (electropherogram). Electrophoresis corresponds to the RT-qPCR products obtained from RNA of a control and MD-392, who is heterozygous for *CPLANE1* c.7588+7A>G. (**B**) Detection and transcript analysis of CPLANE1 c.2747-1981_6172-78del. Copy number variation (CNV) detection based on gene panel data using DECoN (upper image). Visualization plot from DECoN shows the ratio of observed to expected coverage depth of the captured regions with a 95% confidence. Significantly lower scores values suggested a heterozygous deletion from exon 16 to 32 (red dots); DECoN results were validated through CPLANE1 transcript analysis (middle image). RT-PCR was performed on RNA derived from the patient MD-392. Sequencing of the mutated amplicon showed the complete deletion of exons 16 to 32 (electropherogram), which implies a change in the reading frame. Size and genomic breakpoints of the CNV were defined (lower image) by PCR and Sanger sequencing revealed a microhomology region at the breakpoint junction (dash boxed sequence). (**C**) Transcript analysis of *PI4KA* c.3845C>T. Schematic representation of the aberrant splicing; please see description in (**A**). Electrophoresis corresponds to the RT-qPCR products obtained from RNA of a control and MD-610, who is heterozygous for *PI4KA* c.3845C>T. (**D**) Expression analysis of CLK2 p.Y374H. Western-blot analysis of protein extracts obtained from HEK293T cells, transiently transfected with *CLK2* gene wild-type or mutant (p.Y374H), indicates a decreased relative expression of CLK2-mutant compared to the native protein. Three biological replicates are shown. Data is presented as average ± SEM. Significance was determined by two-tailed Student’s *t* test. * *p* < 0.005.

The boy MD-392 (*CPLANE1/C5orf42*, Table 1 and Table 2) was first seen at 18 months of age due to motor delay. He is the only child of healthy parents, with no personal or family history of interest, including neonatal period. Sitting was achieved around 8–9 months. At exam, he presented axial and limb hypotonia without weakness, and patellar hyporeflexia. Independent gait was achieved at 22 months but with instability and a wide base of support. An MRI revealed a hypoplastic cerebellar vermis at the infratentorial level, horizontalized superior cerebellar peduncles, and a dilated fourth ventricle. The pontine midbrain junction took the form of the “molar sign”, all suggestive of JB (Figure 2G). Their IQ was at the lower limit of normal with difficulties in processing speed and attention. The last examination was carried out at the age of 17 where motor difficulties persisted, he was not able to walk in tandem, although with normal gait, and he was capable of running or monopodial jumping. Using the gene panel SPGAtaxia-363, two variants in heterozygosis of *CPLANE1* were detected in MD-392: a previously reported splicing change c.7588+7A>G and a novel deletion c.2747-1981_6172-78del (Table 1). Each parent harbours one of these variants in heterozygosis. We explored the effect of both variants by transcript analysis and concluded that they should cause a deletion of 55 bp causing the skipping of exon 37 (Figure 3A), and a deletion of 45,305 bp causing the lost from exon 16 to 32 (Figure 3B). This large deletion was detected after a CNV (copy number variation) screening using DECoN, which allowed us to detect a significantly lower coverage depth of the *CPLANE1* capture regions spanning exons 16 to 32 (NM_023073.4) compared with the rest of the samples suggesting a heterozygous intragenic deletion in the proband MD-392. By PCR and subsequent Sanger sequencing, we established that the deletion was of 45,305 bp (chr5: 37,170,413-37,215,717; GRCh38) from intron 15 to intron 32. Further transcript analysis showed the absence of exons 16 to 32, suggesting a change in the reading frame and a premature termination codon (NP_075561.3: p.G916Afs*19; Figure 3B). 

The girl MD-297 (*TBCD*, Table 1 and Table 2), born to consanguineous parents (first cousins), was first seen at 15 months due to psychomotor retardation, standing with support, and with corticospinal signs. At the age of 3, she only stood in a standing frame and did not walk even with a walker and presented spasticity and dystonic attitudes to manipulation. Several osteotomies and tenotomies were performed at the ages of 9 and 11. She did not improve with levodopa. With time, she showed bradypsychia and progressive hypomimia, with constant drooling, tetraparesis and loss of head control. A cognitive decline was also ascertained. She also developed acquired microcephaly, precocious puberty at the age of 7, and angiokeratomas from 12 years. Serial neuroimaging showed white matter signal abnormalities that improved overtime but also showed progressive thinning of the corpus callosum, cerebellar ataxia and increasing cortical CA (Figure 2H). At the age of 18, the *TBCD* c.3099C>G (p.N1033K) variant was detected by WES-proband in homozygosis. Her healthy mother carried the *TBCD* c.3099C>G in heterozygosis and her paternal uncle did not (sample from the MD-297′s father was not available). *TBCD* c.3099C>G was previously associated with early-onset progressive encephalopathy (Table 1). TBCD is one of the chaperones involved in the assembly of tubulin heterodimers for their incorporation into microtubules. The three-dimensional structure of this protein is unknown, although it is predicted that it consists of in tandem α helix-turn-α helix repeats arranged in an α solenoid structure (Figure 1C) [23]. In the TBCD tertiary structure modelled by AlphaFold, N1033 is located in the turn between two antiparallel helices, with its side chain oriented towards the protein surface. Although the predictive power of AlphaFold to orient different domains is limited, it is likely that the missense change to lysine results in charge change on the protein’s surface in addition to introducing a bulkier side chain. For both reasons, it is possible to anticipate that this mutation could either affect the interaction between TBCD domains or the interaction of TBCD with other proteins, which could be relevant for the functioning of TBCD given the involvement of this protein in complexes with other tubulin assembly factors [24].

The patient MD-471 (*CLN6*, Table 1 and Table 2), a 7 year-old male, presented with language delay and clumsiness from 3 years of age. At 5 years old, a neurological examination revealed dysarthria, drooling, gait instability, and occasional drop attacks. An EEG disclosed generalised polyspike wave complexes and slow background activity. A brain MRI showed global cerebral and cerebellar atrophy, and white matter hyperintensities (Figure 2I,J). The clinical course was rapidly progressive from age 6, and at 7 years, he was unable to walk alone and presented with severe visual deficit, loss of speech and auditive-triggered myoclonus. WES-proband showed that the patient was compound heterozygote for c.214G>T (p.E72*) and c.829_836delinsCCT (p.V277Pfs*5), in *CLN6*. Both variants have been before related to disease (Table 1). The further segregation analysis showed that each parent carried one of these mutations, and that his younger sister also was compound heterozygous, as her brother. His sister was included in the genetic study because of mild stuttering from the age of 3. At that age, her brain MRI already showed enlarged cerebellar folia interspaces and white matter hyperintensities. In the subsequent year, gait instability, dysarthria and cognitive decline symptoms appeared in the sister.

The patient MD-610 (*PI4KA*, Table 1 and Table 2), presented at 20 months of age with acute gait instability after being treated with amoxicillin because of a middle ear infection. A post-infectious cerebellar syndrome was suspected, but a brain MRI revealed predominantly supratentorial T2 diffuse myelin hyperintensity suggesting hypomyelination, with mild enlargement of folia interspaces in both cerebellar hemispheres (Figure 2K). From 2 to 3 years of age, he developed progressive spasticity in lower limbs, with cognitive and language preservation. A control MRI at 30 months of age confirmed hypomyelination and CA. The genetic analysis by WES-trio detected that the patient was compound heterozygote for c.3845C>T (p.A1282V) and c.2750T>C (p.F917S) in *PI4KA*. Both mutations are novel and each one was inherited for a parent (Table 1). *PI4KA* c.3845C>T was predicted as a splicing mutation and a transcript analysis was performed (Figure 3C) that revealed that should cause a deletion of 57 bp causing partial skipping of exon 33. 

Finally, the patient MD-436 (*CLK2*, Table 1 and Table 2), was first seen at 17 months of age due to motor delay. She was able to walk with support but could not stand from sitting by herself. At exam, she presented with hypotonia, and a limitation to move her eyes side-to-side, shaking the head to the direction of interest. Further exams were consistent with oculomotor apraxia with mild improvement over age. Some dysmorphic features were ascertained: small orbits, pointed palate and small hands. A CGH-array did not reveal any relevant CNV. A brain MRI showed a subtle enlargement of vermian folia interspaces at 20 months of age. At 5 years, she could walk independently but presented some difficulties in tandem. School performance and expressive language were below average but improved with time. A de novo novel variant was detected by WES-trio in heterozygosis in the proband, c.1120T>C (p.Y374H), in *CLK2*. No other variants of interest were detected. To investigate the structural effects of CLK2 p.Y374H, we used HOPE that determined the disruption of the hydrogen bond formed between the residue Y374 and the threonine at position 250, and Foldx that predicted a notable structural destabilization of the mutated protein and calculated a 4.00776 kcal/mol change in free energy. CLK2 is a dual specificity kinase that phosphorylates splice factors acting on both serine/threonine and tyrosine-containing substrates. To be active, CLK2 first goes under auto-phosphorylation of its tyrosine residues; then, it phosphorylates serine- and arginine-rich (SR) proteins of the spliceosomal complex. The proband has mutated the residue Y374. To test whether the detected p.Y374H affects the CLK2 protein levels suggesting instability of the mutated protein, as previously described for the variants p.K192R and p.T343A in mice [25,26], we transfected transiently the mutated protein in HEK293T cells. A significantly decreased expression of CLK2-mutant compared to the native protein was appreciated (Figure 3D), which may indicate a deficient activity of CLK2-Y374H. 

## 3. Discussion

The number of disorders that present with CA is ever-increasing with an extremely broad clinical spectrum, from non-progressive developmental disorders to neurodegenerative conditions [7,9,10,11]. Pure CA, defined as an isolated CA based on neuroimaging, is commonly found in SCAs and SCARs, but also in some inborn errors of metabolism and mitochondrial disorders [9,27]. CA may present with additional supratentorial findings such as hypomyelination, periventricular progressive matter abnormalities, and basal ganglia involvement, which would add clinical value for a pattern-recognition approach [6,27]. We report here ten new cases of early-onset cerebellar involvement with a great clinical and genetic heterogeneity. 

In this cohort, CA, as determined in serial neuroimaging, is present in eight individuals with variants in 7 genes: *SETX*, *CACNA1G*, *CACNA1A*, *MAST1*, *TBCD*, *CLN6*, and *PI4KA*, highlighting the heterogeneity of associated genotypes with a clear progression of CA over time. Additionally, in three patients a white matter involvement was associated with CA carrying mutations in *CLN6*, *MAST1* and *PI4KA* genes. A suggestive MRI pattern was identified in two patients with mutations in *MAST1*, associated with pseudofusion of the putamen and caudate, and mega-corpus callosum size, and in *TBCD*, causing hypomyelination, and CA and callosal thinning, which are hallmarks of this progressive disorder.

So far, 338 different variants in *SETX* are annotated in HGMD^®^ (Human Genetic Mutation Database Professional 2023.1; accessed on 14 September 2023), some of them especially recurrent, as the mutation identified in homozygosis in the girl MD-359, c.5825T>C. Modelling of the protein showed that the mutation may destabilize the folding affecting its stability. Mutations in *SETX* transmitted in an AD manner are involved in amyotrophic lateral sclerosis-4 (ALS4), and with an AR mode of inheritance, are responsible for SCA with axonal neuropathy (SCAN2), which is characterised by progressive CA, along with sensorimotor neuropathy, occasional oculocephalic dissociation and/or oculomotor apraxia, strabismus, chorea, dystonia, and elevated serum α-fetoprotein levels [28,29]. Polyneuropathy is present in 97.5% of patients suffering from *SETX*-associated ataxia and CA in 96% [29]. Senataxin, which contains a DNA/RNA helicase domain, participates in the DNA repair pathway and mutations in *SETX* may cause neuronal degeneration through dysfunction of helicase activity or other steps in RNA processing [30,31]. MD-359 was a Roma girl born to consanguineous parents. She showed a mild clinical picture with mild-moderate CA and unsteady gait together with increased levels of α-fetoprotein (13.6 µg/L). She also presented with moderate bilateral sensory hearing loss, which might not be linked to *SETX*. 

Implication of ion channels in genetic ataxias associated with CA is long known, given the importance of ion balance, notably that of calcium ions, in cerebellar pathology. In fact, in mutational screening of clinical series of patients with cerebellar ataxia, the frequency of channelopathies is relatively high [32,33]. This group of disorders comprises a large number of clinically heterogeneous diseases usually comprising epilepsy. Damaging variants in *CACNA1G* leads to SCA42, a slowly progressive ataxia, characterised by severe motor, cognitive impairment, and CA, with variable features such as facial dysmorphisms, digital anomalies, microcephaly, and epilepsy [5,34]. CACNA1G encodes the low-voltage-activated Ca(v)3.1 T-type calcium channel, and highly expressed in Purkinje neurons and deep cerebellar nuclei; mutations in CACNA1G may result in decreased neuronal excitability [5,34]. MD-353 harbours a *de novo CACNA1G* c.2881 G>A change in heterozygosis, a well-known mutation associated with SCA42. Modelling of the mutated protein showed that the change p.A961T may affect the intracellular segment required for binding to the β regulatory subunit of this channel, which may impair the proper functioning of the channel. MD-353 is at present, a 17 year-old boy, with a stable ataxia course and no epilepsy, who at age of 13 months displayed cerebellar reduced volume that was associated with CA afterwards. He showed moderate cognitive impairment and attention deficit. Strikingly, the proband suffered from congenital arthrogryposis, which is not a common feature related to ataxias [35], although it is for instance, associated with pontocerebellar hypoplasia type 1A due to *VRK1* mutations that occasionally includes ataxia [36]. 

Another more frequent ataxia due to defects in a calcium channel is SCA6, caused by mutations in *CACNA1A*. Episodic ataxia type 2 (EA2), developmental and epileptic encephalopathy 42 (DEE42), and familial hemiplegic migraine are also due to *CACNA1A* variants. EA2 or SCA6 can be caused by abnormal (CAG)_n_ expansions as well as point mutations. The *CACNA1A*-associated phenotypic spectrum is really much more wider comprising early developmental delay, autism spectrum disorders, epileptic encephalopathy, and early onset paroxysmal dystonia [37], as revealed by the detection of mutations facilitated by the use of NGS-based genetic diagnostic tools. In fact, more than 400 mutations in *CACNA1A* are known (HGMD^®^ Professional 2023.1; accessed September 14, 2023). The *CACNA1A* gene encodes the transmembrane pore-forming subunit of the P/Q-type or CaV2.1 voltage-gated calcium channel, which functions as a transcription factor that mediated cerebellar development [38]. The *CACNA1A* probands carry c.3042C>G (MD-309) or c.234_235delCT (MD-556), in heterozygosis, which have not been previously associated with disease. 

Neuronal ceroid lipofuscinosis (NCLs) are a group of inherited lysosomal storage disorders with a notable genetic heterogeneity (at least 15 genes involved) and a complex phenotype. Deleterious mutations in *CLN6* lead to NCL type 6A and type 6B. *CLN6* encodes for a transmembrane protein that is expressed in in adult and embryonic brain and in peripheral tissues of mouse and human [39]. CLN6 dysfunction leads to changes in the storage material, which consists of autofluorescent lipopigments, in the lysosomes causing the destruction and loss of neurons in the cerebral, cerebellar cortex and retina [40]. The clinical picture comprises intellectual and motor deterioration, visual impairment, seizures, psycho-motor decline, and loss of neurons [41]. The *CLN6*-associated phenotype fits with this clinical picture and fatal outcome with premature death. Ataxia is a common additional neurological sign, and even may be the primary clinical feature at symptoms onset, which may mislead the diagnosis of NCL [42]. Other frequent manifestations include developmental regression, seizure, and ID [43]. Patient MD-471 fits completely with the *CLN6*-associated phenotype, including CA and abnormal cerebral white matter morphology, which are also observed in CLN6 patients [43]. Both stop mutations in CLN6, p.E72* and p.V277Pfs*5, described in MD-471 and his sister have been reported in several patients (HGMD^®^ Professional 2023.1; accessed on 14 September 2023). 

*MAST1* alterations lead to a mega-corpus-callosum syndrome with cerebellar hypoplasia and cortical malformations. The clinical picture comprises non-verbal moderate to profound ID, and hypotonia in all of them [44]. To our knowledge, eleven cases are known caused by seven different mutations in *MAST1* (HGMD^®^ Professional 2023.1; accessed on 14 September 2023). McMichael et al. [45] reported a *de novo* novel missense mutation in a patient with diplegic cerebral palsy, but with no further clinical information. Tripathy et al. [46] delineated the clinical picture described above, with six patients presenting the neuroimaging triad, associated with non-verbal ID and hypotonia in all of them. Additionally, two patients also presented with seizures and two with oculomotor apraxia, and a mild truncal ataxia was described in one patient with a frameshift variant. Few cases with *MAST1* mutations have been reported with atypical presentations, such as absence of cerebellar hypoplasia, predominant bilateral polymicrogyria, dysmorphic features, short stature, epileptic encephalopathy, and hypogonadotropic hypogonadism [47,48,49,50]. The c.1672G>C (p.V558L) variant in MAST1 identified in MD-548 is a *de novo* novel change predicted as damaging according to the ACMG/AMP criteria [20]. MD-548 presents with the main clinical signs associated with the neurodevelopmental disorder caused by *MAST1* mutations. He is currently a nonverbal teen with a severe ID, hypotonia, and truncal ataxia, but a cortical development malformation is lacking in neuroimaging. Additionally, it was disclosed a bilateral thinning of the anterior arm of the internal capsule resembling a pseudo-fusion of putamen and caudate nuclei, not previously reported. With MD-548, we describe the novel *MAST1* c.1672G>C mutation, expanding the clinical picture of this ultrarare neurodevelopmental disorder, suggesting that ataxia may be more frequently present but masked as persistent hypotonia in other cases. 

The proband MD-392 was a compound heterozygous for c.7588+7A>G and c.2747-1981_6172-78del in *CPLANE1*. Both mutations investigated by transcript analysis revealed that altered the proper splicing causing the loss of exons. Damaging variants in *CPLANE1* can cause JS or more frequently, orofaciodigital syndrome VI (OFD6). Around 35 genes are involved in JS and related disorders, which are characterised by a pathognomonic cerebellar and brainstem malformation on brain MRI, the so-called molar tooth sign. JS is a multisystem syndrome with a complex phenotype that affect different body systems (eyes, kidney, liver and skeleton), clinically characterised by hypotonia, ataxia, developmental delay or ID, oculomotor apraxia, and apnea or hyperapnea [51]. MD-392 displayed a pure neurological form of JS with borderline intelligence and no retinal or kidney involvement, although he occasionally presented with oculomotor apraxia, together with hypotonia and molar tooth sign on MRI. The *CPLANE1* (ciliogenesis and planar polarity effector) protein is required for cilium assembly in ciliated cells [52]. Abnormal cell cycle regulation and DNA damage signalling are involved in the pathogenesis of ciliopathies [53,54]. Upon entry into mitosis, JBTS17 is localised to the kinetochore; a knockdown of Jbts17 causes mitotic arrest of progenitor cells and defects in neuronal migration in cerebral cortex in developing mouse brain [52].

Early-onset progressive encephalopathy with brain atrophy and a thin corpus callosum (PEBAT) is caused by damaging variants transmitted in an AR fashion in TBCD. TBCD is one of the five tubulin-specific chaperones (TBCA-E) needed for the reversible assembly of the α-/β-tubulin heterodimers, which are involved in crucial cell functions such as division or intracellular transport [55]. Patient fibroblasts showed altered microtubule dynamics, supporting that microtubule dynamic has an impact on neuronal function and survival in the brain [23]. TBCD depletion may result in an abnormal microtubule network and abnormal microtubule trafficking of mitochondria in the human brain, leading to decreased energy supply to neuronal cells and subsequent neuronal degeneration. PEBAT is characterised by a broad clinical spectrum even between siblings [55,56]. The girl, MD-297, presented with a severe neurological dysfunction with encephalopathy, spastic-dystonic tetraparesis, and severe ID. The observed neuroimaging findings resembled those usually associated with PEBAT: hypomyelination, CA and callosal thinning. The TBCD p.N1033K mutation identified in homozygosis in the patient MD-297 was reported as a founder event in the Faroe Islands [57]; to our knowledge, MD-297 and their relatives were all from Spain. The structural modelling of TBCD p.N1033K predicted that its capacity for interacting with other proteins may be impaired, which may affect its proper activity given the involvement of TBCD in complexes other tubulin assembly factors [24]. 

AR mutations in *PI4KA* cause a variable phenotypical spectrum ranging from severe neurodevelopmental disorder with spasticity, hypomyelinating leukodystrophy, and brain abnormalities (NEDSPL) to pure spastic paraplegia type 84 (SPG84) [58]. Additionally, patients can present with inflammatory bowel disease, multiple intestinal atresia, and combined immunodeficiency [59]. MD-610 presented with ataxia from 2 years of age, but soon developed spasticity in lower limbs. At 3 years of age, cognitive and language development were within normal limits, and he could walk alone. A brain MRI showed diffuse alterations of the pattern of supratentorial myelination and incipient cerebellar atrophy involving hemispheres. MD-610 carried novel biallelic variants in *PI4KA* and the transcript analysis performed for c.3845C>G revealed that may affect the proper splicing. Two patients have been previously described with a similar clinical picture without cognitive or language impairment, but both did not show supratentorial white matter hypomyelination as ours, so a longer follow-up must be needed to ascertain the milder phenotype [58]. 

The proband MD-436 suffered from a mild ataxia, oculomotor apraxia, borderline IQ, and language delay, with global improvement over time. A single brain MRI revealed a reduced vermian volume with enlarged interfolia spaces at two years of age. In the patient MD-436 investigated by WES-trio, the only variant of interest was the *de novo* novel variant *CLK2* c.1120T>C (p.Y374H) in heterozygosis. To date, no Mendelian disorders have been associated with *CLK2* mutations. A translocation t(1;19) (q21.3;q13.2), affecting *CLK2* and *PAFAH1B3*, was reported in a female with ID, ataxia and brain atrophy [60]. The molecular mechanism was associated with *PAFAH1B3*, which resulted to be inactive and hence, without capacity of interacting with LIS1 (related to lissencephaly), a member of the heterotrimeric G protein complex PAF-AH1B. CLKs belong to the dual-specificity protein kinase family and play an essential role in the regulation of the transcript splicing via the phosphorylation of the serine/arginine-rich (SR) proteins and subsequently modulates the alternative splicing of precursor mRNA (pre-mRNA) [61]. The patient MD-436 had a mutated residue of tyrosine, Y374. CLK2 protein level is regulated by CLK2 kinase activity [25]. The CLK2-K192R and -T343A mutants were demonstrated to be less stable and with a reduced protein expression [25]. We have demonstrated that CLK2-Y374H shows a decreased expression that may reflect a reduction in the kinase activity of the mutated protein due to its instability, predicted by the in silico tools HOPE and FoldX, which possibly affects its substrate interaction properties. Levels of phosphorylation of substrates correlates with the CLK2 expression. PGC-1α, a CLK2 substrate, is a widely expressed transcriptional regulator, which modulates the expression of genes engaged in mitochondrial biosynthesis, metabolic regulation, and oxidative stress [62,63]. Recent studies have demonstrated that PGC-1α protects against neuroinflammation and performs a neuroprotective influence in neurodegenerative diseases and, therefore, plays a critical role in the pathophysiology of many neurodegenerative disorders [62,63], which may participate in the clinical presentation of a neurodevelopmental disorder with ataxia and oculomotor apraxia. Further studies are needed to elucidate if CLK2 p.Y374H is the causative defect leading to the clinical presentation in our patient or is a change that partly contributes to the clinical picture. 

## 4. Patients and Methods

### 4.1. Patients and Clinical Features

Ten probands were selected based on the presence of CA associated to ataxia, ID and/or additional neurological signs and symptoms. Phenotyping included standardised assessments of ataxia, movement disorders, and other neurological symptoms such as spasticity, muscle weakness or signs of peripheral neuropathy. An intelligent quotient was obtained by WISC-IV [64]. MRI studies were performed in 1.5- and 3-T scanners, depending on the referral hospitals. CA was ascertained by loss of cerebellar volume in at least two consecutive cerebral MRI studies [9]. 

### 4.2. Genetic and Genomic Studies

Eight out of ten patients went for WES-proband or WES-trio, depending on the samples sequenced) based on a xGen Exome Research Panel with custom-designed capture probes (Integrated DNA Technologies, Coralville, IA, USA) at Blueprint Genetics (Helsinki, Finland) or KAPA HyperExome (Roche, Basel, Switzerland) at CNAG (Centre Nacional d’Anàlisi Genòmica, Barcelona, Spain). MD-309 and MD-392 were investigated using a custom panel for ataxia and spastic paraplegia (Panel SPGAtaxia-363) based on SureSelect Custom Panel (Agilent Technologies, Santa Clara, CA, USA) including 363 genes (gene list upon request). 

Bioinformatics analyses (filtering data, study of the novelty and pathogenicity of the candidate variants and CNVs, copy number variants) were carried out in-home as previously described [65]. To classify the identified variants according to its pathogenicity, we followed the ACMG/AMP criteria [20].

Sanger sequencing on an ABI Prism 3130XL analyser (Applied Biosystems, Foster City, CA, USA) was performed for validation by PCR and segregation analysis of the genetic candidate variants identified in the bioinformatics analysis. Primers are available upon request.

Copy number variants (CNVs) were investigated using the coverage-based DECoN bioinformatics tool. BAM files from samples with consistent exon CNVs calls were visually inspected in the Integrative Genomics Viewer (IGV) to find additional support for the calls and putative breakpoints, that were further determined by PCR and Sanger sequencing of DNA samples. 

### 4.3. Structural Modelling of SETX p.I1942T, CACNA1G p.A961T and TBCD p.N1033K

The structural analysis of SETX was based on the experimental structure of the yeast SEN1 helicase domain (PDB entry 5mzn) [19], as well as on the region corresponding to this domain in the model generated by AlphaFold for human SETX (accession code Q7Z333) [66,67]. In the absence of experimental structural information for TBCD, the structural analysis for this protein was based on a model generated by AlphaFold (https://aphafold.com; accession code Q9BTW9) [66,67]. For CACNA1G we used the structure of the transmembrane region of the human protein (PDB entries 6zzp and 6kzo; Zhao et al. 2019) as well as the structure of the voltage-gated calcium channel Cav1.1 complex from rabbit (PDB entry 5gjv) [22], where some of the cytoplasmic elements not visible in the human CACNA1G structure, in addition to the regulatory proteins, are included. Figure 2 was prepared using PYMOL Molecular Graphics System, v2.0 Schrödinger, LLC (https://pymol.org/2/).

### 4.4. Transcript Analysis of CPLANE1, c.7588+7A>G and c.2747-1981_6172-78del, and PI4KA, c.3845C>T

For further investigation of variants detected in *CPLANE1,* c.7588+7A>G and c.2747-1981_6172-78del, as well as the variant c.3845C>T identified in *PI4KA* (Table 1), transcript analyses were performed as previously described [65]. Table S1 lists the used primers. 

### 4.5. In Silico Analyses and Protein Expression Levels of CLK2 p.Y372H

FoldX [68] was used to calculate the variation in folding free energy of the mutations with respect to the WT (ΔΔG). Higher values correlate with less stable proteins when mutated. The web-based platform HOPE [69] was employed to evaluate differences in physicochemical properties and disruptions of protein interactions induced by mutated residues.

The CLK2 cDNA (NM_001294338.2) cloned in the pcDNA3.1+/C-(K)-DYK vector (oHu28699D), tagged with DYK in C-ter, was obtained from GenScript (Piscataway, NJ, USA). The CLK2 p.Y374H mutation was generated with specific primers (Table S1) using the QuikChange Site-Directed Mutagenesis Kit (Agilent, Santa Clara, CA, USA). HEK293T cells were transiently transfected for 48 h with 1 µg of WT (wild-type) or mutated CLK2 and FuGENE HD Transfection Reagent (Promega, Madison, WI, USA). Whole cell protein extraction with lysis buffer supplemented with the phosphatase inhibitor cocktail PhosSTOP™ (Roche Diagnostics GmbH, Mannheim, Germany), SDS-PAGE and western blotting were performed as previously described [8]. Primary antibody dilutions used in western-blot were as follows: anti-FLAG M2^®^ (Sigma-Aldrich, St. Louis, MO, USA): 1/1000 and anti-GAPDH (0411) (Santa Cruz Biotechnology, Dallas, TX, USA): 1/8000. 

## 5. Conclusions

New genes and new variants are constantly identified thanks to the increased availability of exome and genome sequencing together with new methods such as long-read sequencing. To determine if a genetic variant is deleterious or not is essential. Moreover, most conditions are rare disorders characterised by genetic heterogeneity and allelic diseases. Definitely, diagnosis is a challenge. Therefore, the characterisation of new cases that support the known phenotypes, reveal new clinical pictures, and/or show new diseases due to genes not yet associated with Mendelian diseases is crucial to improve the genetic diagnosis, mainly in childhood. In this work, we contributed to the landscape of early-onset cerebellar atrophy with ten new cases and nine different genes, including additional experiments to gain evidence of the pathogenicity of several variants.

## Figures and Tables

**Table 1:** Genetic findings.

Patient	Gene	RefSeq	Position ^†^	DNA Change	Protein Change	Prediction ^‡^	rs Number (MAF) ^§^	References PMID ^‖^	Method
MD-359	*SETX*	NM_015046.7 NP_055861.3	chr9:132297011	c.5825T>C HOMOZYGOSIS (Consanguinity)	p.I1942T	P	rs773379832 (0.00001195)	27549087 35872528 33098801 27528516 34426522	WES-proband
MD-353	*CACNA1G*	NM_018896.5 NP_061496.2	chr17:50592063	c.2881G>A	p.A961T	P	rs886041505 (0)	29878067 33098379 30842224 32736238 31785789 31836334	WES-proband
MD-309	*CACNA1A*	NM_001127221.2 NP_001120693.1	chr19:13298594	c.3042C>G	p.Y1014*	P	Novel	Novel	Gene panel
MD-556	*CACNA1A*		chr19:13505990-13505991	c.234_235delCT	p.F79Pfs*22	P	rs762483619 (0.000004023)	NA	WES-trio
MD-471	*CLN6*	NM_017882.3 NP_060352.1	chr15:68214373	c.214G>T	p.E72*	P	rs104894483 (0.00006761)	11791207 35012600 31589614 35505348 25525159	WES-proband
			chr15:68208244-68208248	c.829_836delinsCCT	p.V277Pfs*5	P	rs1595816474 (NA)	34440436 35505348 12815591	
MD-548	*MAST1*	NM_014975.3 NP_055790.1	chr19:12865349	c.1672G>C	p.V558L	P	Novel	Novel	WES-trio
MD-392	*CPLANE1*	NM_023073.3 NP_075561.3	chr5:37164266	c.7588+7A>G	p.E2512Kfs*18	P	rs773662834 (0.00002389)	28431631	Gene panel
			chr5:37170413-37215717	c.2747-1981_6172-78del	p.G916Afs*19	P	Novel	Novel	
MD-297	*TBCD*	NM_005993.5 NP_005984.3	chr17:82930629	c.3099C>G HOMOZYGOSIS (No consanguinity)	p.N1033K	P	rs748615072 (0.000007128)	29921875	WES-proband
MD-610	*PI4KA*	NM_058004.4 NP_477352.3	chr22:20734450	c.3845C>T	p.A1282_D1300del	LP	Novel	Novel	WES-trio
			chr22:20761345	c.2750T>C	p.F917S	LP	Novel	Novel	
MD-436	*CLK2*	NM_001294338.2 NP_001281267.1	chr1:155264494	c.1120T>C	p.Y374H	LP	Novel	Novel	WES-trio

LP, likely pathogenic; NA, not available; P, pathogenic; PMID, PubMed identifier; RefSeq, reference sequence; WES, whole exome sequencing; MAF, minor allele frequency ^†^ Chromosome position based on GRCh38. ^‡^ Prediction of pathogenicity performed according to the ACMG/AMP guidelines [20]. ^§^ Consulted database gnomAD v2.1.1 (accessed on 27 March 2023). ^‖^ References associated with each mutation in the Human Mutation Data Base (HGMD^®^) Professional 2023.1 (accessed on 14 September 2023).

**Table 2:** Clinical features.

Patient Sex	Gene	Presentation Inheritance	Disease (OMIM)	Age of Onset Age at Testing	Clinical Presentation	Brain MRI	Cognitive Involvement	Other Neurological Phenotypic Characteristics
MD-359 female	*SETX*	Sporadic AR	SCA with axonal neuropathy (606002)	6 yo 8 yo	Mild truncal ataxia. Bilateral sensorineural deafness.	CA	Average IQ	Learning difficulties
MD-353 male	*CACNA1G*	*de novo* AD	SCA42 (616795) SCA42 early-onset, severe, with neurodevelopmental deficits (618087)	At birth 17 yo	Congenital arthrogryposis. Ataxia. Gait instability. Dysmetria.	Moderate CA	Moderate Attention deficit	Moderate-severe speech delay. Dystonic head movements. Nystagmus
MD-309 male	*CACNA1A*	*de novo* AD	Episodic Ataxia type 2 (108500) SCA6 (183086) Developmental and epileptic encephalopathy 42 (617106) Familial hemiplegic migraine type 1 (141500)	18 mo 12 yo	Gait instability. Basal nystagmus. Dysmetria. Episodes of increased instability that last hours.	Mild vermian atrophy	Mild IQ	Arreflexia
MD-556 female	*CACNA1A*	*de novo* AD		6 mo 12 yo	Paroxysmal, episodic, vertigo-like disorder triggered by emotions or fever. Gait instability.	Mild vermian atrophy	Borderline IQ	Strabismus. Mild gait ataxia. EEG abnormalities
MD-471 male	*CLN6*	Familial AR	Neuronal Ceroid Lipofuscinosis Type 6A (601780) Type 6B (204300)	3 yo 5 yo	Dysarthria. Gait instability. ADHD symptoms.	CA White matter T2/FLAIR hyperintensity	Progressive decline	Drop attacks. Visual deficit and gait loss from 6-yo.
MD-472 ^†^ female				3 yo 3 yo	Mild stuttering.	Enlarged cerebellar folia, white matter T2/FLAIR hyperintensity	Mild attention deficit	Gait instability, loss of speech and cognitive decline from 4-yo.
MD-548 male	*MAST1*	*de novo* AD	Mega-corpus-callosum syndrome with cerebellar hypoplasia and cortical malformations (618273)	At birth 13 yo	Ataxia. Lack of speech.	Megacorpus callosum with CA	Severe ID	Absence of language. Mild facial dysmorphic features. Tented upper lip, pointed palate
MD-392 male	*CPLANE1*	Sporadic AR	Joubert syndrome 17 (614615)	18 mo 17 yo	Motor delay (independent walk 2 yo).	Molar tooth sign. Cerebellar hypoplasia	Borderline IQ	Ataxia. Oculomotor apraxia. ADHD.
MD-297 female	*TBCD*	Sporadic AR	Early-onset progressive encephalopathy with brain atrophy and thin corpus callosum (PEBAT) (617193)	15 mo 18 yo	Seizures. Motor impairment.	Progressive CA	Severe ID	Encephalopathy Spastic-dystonic tetraparesis
MD-610 male	*PI4KA*	Sporadic AR	Neurodevelopmental disorder with spasticity, hypomyelinating leukodystrophy, and brain abnormalities (NEDSPLB) (616531)	20 mo 36 mo	Acute ataxia.	Diffuse hypomyelination Enlarged cerebellar folia	No	Progressive lower limbs spasticity
MD-436 female	*CLK2*	*de novo* AD	-	17 mo 5 yo	Motor delay. Gait instability. Oculomotor apraxia.	Subtle enlargement of vermian folia	Borderline IQ	Global improvement over age. Language delay

OMIM, Online Mendelian Inheritance in Men; MRI, magnetic resonance imaging; FLAIR, fluid-attenuated inversion recovery; EEG: electroencephalogram; AR, autosomal recessive; AD, autosomal dominant; mo, months old; yo, years old; SCA, spinocerebellar ataxia; ADHD, attention deficit hyperactivity disorder; IQ, intellectual quotient; ID, intellectual disability; CA, cerebellar atrophy. ^†^ MD-472 is sister of proband MD-471.

## Data Availability

Data are contained within the article.

## Supplementary Materials

The following are available online at https://www.mdpi.com/article/10.3390/ijms242216400/s1.

## References

[1] Ataullah A.H.M., Naqvi I.A. (2023). Cerebellar Dysfunction.

[2] Schmahmann J.D. (2018). The cerebellum and cognition. Neurosci. Lett..

[3] Tavano A., Grasso R., Gagliardi C., Triulzi F., Bresolin N., Fabbro F., Borgatti R. (2007). Disorders of cognitive and affective development in cerebellar malformations. Brain.

[4] Al-Maawali A., Blaser S., Yoon G. (2012). Diagnostic approach to childhood-onset cerebellar atrophy: A 10-year retrospective study of 300 patients. J. Child Neurol..

[5] Chemin J., Siquier-Pernet K., Nicouleau M., Barcia G., Ahmad A., Medina-Cano D., Hanein S., Altin N., Hubert L., Bole-Feysot C. (2018). *De novo* mutation screening in childhood-onset cerebellar atrophy identifies gain-of-function mutations in the CACNA1G calcium channel gene. Brain.

[6] Poretti A., Wolf N.I., Boltshauser E. (2015). Differential diagnosis of cerebellar atrophy in childhood: An update. Neuropediatrics.

[7] Sancho P., Andrés-Bordería A., Gorría-Redondo N., Llano K., Martínez-Rubio D., Yoldi-Petri M.E., Blumkin L., de la Fuente P.R., Gil-Ortiz F., Fernández-Murga L. (2021). Expanding the β-III spectrin-associated phenotypes toward non-progressive congenital ataxias with neurodegeneration. Int. J. Mol. Sci..

[8] Martínez-Rubio D., Rodríguez-Prieto Á., Sancho P., Navarro-González C., Gorría-Redondo N., Miquel-Leal J., Marco-Marín C., Jenkins A., Soriano-Navarro M., Hernández A. (2022). Protein misfolding and clearance in the pathogenesis of a new infantile onset ataxia caused by mutations in *PRDX3*. Hum. Mol. Genet..

[9] Martínez-Rubio D., Hinarejos I., Sancho P., Gorría-Redondo N., Bernadó-Fonz R., Tello C., Marco-Marín C., Martí-Carrera I., Martínez-González M.J., García-Ribes A. (2022). Mutations, genes, and phenotypes related to movement disorders and ataxias. Int. J. Mol. Sci..

[10] Darling A., Aguilera-Albesa S., Tello C.A., Serrano M., Tomás M., Camino-León R., Fernández-Ramos J., Jiménez-Escrig A., Poó P., O’Callaghan M. (2018). PLA2G6-associated neurodegeneration: New insights into brain abnormalities and disease progression. Park. Relat. Disord..

[11] Tello C., Darling A., Lupo V., Pérez-Dueñas B., Espinós C. (2017). On the complexity of clinical and molecular bases of neurodegeneration with brain iron accumulation. Clin. Genet..

[12] Coarelli G., Coutelier M., Durr A. (2023). Autosomal dominant cerebellar ataxias: New genes and progress towards treatments. Lancet Neurol..

[13] Rossi M., Perez-Lloret S., Doldan L., Cerquetti D., Balej J., Millar Vernetti P., Hawkes H., Cammarota A., Merello M. (2014). Autosomal dominant cerebellar ataxias: A systematic review of clinical features. Eur. J. Neurol..

[14] Synofzik M., Puccio H., Mochel F., Schöls L. (2019). Autosomal recessive cerebellar ataxias: Paving the way toward targeted molecular therapies. Neuron.

[15] Rossi M., Anheim M., Durr A., Klein C., Koenig M., Synofzik M., Marras C., van de Warrenburg B.P., International Parkinson and Movement Disorder Society Task Force on Classification and Nomenclature of Genetic Movement Disorders (2018). The genetic nomenclature of recessive cerebellar ataxias. Mov. Disord..

[16] Sancho P., Sánchez-Monteagudo A., Collado A., Marco-Marín C., Domínguez-González C., Camacho A., Knecht E., Espinós C., Lupo V. (2017). A newly distal hereditary motor neuropathy caused by a rare AIFM1 mutation. Neurogenetics.

[17] Sancho P., Bartesaghi L., Miossec O., García-García F., Ramírez-Jiménez L., Siddell A., Åkesson E., Hedlund E., Laššuthová P., I Pascual-Pascual S. (2019). Characterization of molecular mechanisms underlying the axonal Charcot–Marie–Tooth neuropathy caused by MORC2 mutations. Hum. Mol. Genet..

[18] Abbott J.A., Meyer-Schuman R., Lupo V., Feely S., Mademan I., Oprescu S.N., Griffin L.B., Alberti M.A., Casasnovas C., Aharoni S. (2018). Substrate interaction defects in histidyl-tRNA synthetase linked to dominant axonal peripheral neuropathy. Hum. Mutat..

[19] Leonaitė B., Han Z., Basquin J., Bonneau F., Libri D., Porrua O., Conti E. (2017). Sen1 has unique structural features grafted on the architecture of the Upf1-like helicase family. EMBO J..

[20] Richards S., Aziz N., Bale S., Bick D., Das S., Gastier-Foster J., Grody W.W., Hegde M., Lyon E., Spector E. (2015). Standards and guidelines for the interpretation of sequence variants: A joint consensus recommendation of the American College of Medical Genetics and Genomics and the Association for Molecular Pathology. Anesthesia Analg..

[21] Zhao Y., Huang G., Wu Q., Wu K., Li R., Lei J., Pan X., Yan N. (2019). Cryo-EM structures of apo and antagonist-bound human Ca(v)3.1. Nature.

[22] Wu J., Yan Z., Li Z., Qian X., Lu S., Dong M., Zhou Q., Yan N. (2016). Structure of the voltage-gated calcium channel Cav1.1 at 3.6 Å resolution. Nature.

[23] Flex E., Niceta M., Cecchetti S., Thiffault I., Au M.G., Capuano A., Piermarini E., Ivanova A.A., Francis J.W., Chillemi G. (2016). Biallelic mutations in TBCD, encoding the tubulin folding cofactor D, perturb microtubule dynamics and cause early-onset encephalopathy. Am. J. Hum. Genet..

[24] Fanarraga M.L., Bellido J., Jaén C., Villegas J.C., Zabala J.C. (2010). TBCD links centriologenesis, spindle microtubule dynamics, and midbody abscission in human cells. PLoS ONE.

[25] Rodgers J.T., Haas W., Gygi S.P., Puigserver P. (2010). Cdc2-like kinase 2 is an insulin-regulated suppressor of hepatic gluconeogenesis. Cell Metab..

[26] Nayler O., Schnorrer F., Stamm S., Ullrich A. (1998). The cellular localization of the murine serine/arginine-rich protein kinase CLK2 is regulated by serine 141 autophosphorylation. J. Biol. Chem..

[27] Poretti A., Wolf N.I., Boltshauser E. (2008). Differential diagnosis of cerebellar atrophy in childhood. Eur. J. Paediatr. Neurol..

[28] Renaud M., Tranchant C., Koenig M., Anheim M. (2020). Autosomal recessive cerebellar ataxias with elevated alpha-fetoprotein: Uncommon diseases, common biomarker. Mov. Disord..

[29] Anheim M., Monga B., Fleury M., Charles P., Barbot C., Salih M., Delaunoy J.P., Fritsch M., Arning L., Synofzik M. (2009). Ataxia with oculomotor apraxia type 2: Clinical, biological and genotype/phenotype correlation study of a cohort of 90 patients. Brain.

[30] Moreira M.-C., Klur S., Watanabe M., Németh A.H., Le Ber I., Moniz J.-C., Tranchant C., Aubourg P., Tazir M., Schöls L. (2004). Senataxin, the ortholog of a yeast RNA helicase, is mutant in ataxia-ocular apraxia 2. Nat. Genet..

[31] Chen Y.-Z., Bennett C.L., Huynh H.M., Blair I.P., Puls I., Irobi J., Dierick I., Abel A., Kennerson M.L., Rabin B.A. (2004). DNA/RNA helicase gene mutations in a form of juvenile amyotrophic lateral sclerosis (ALS4). Am. J. Hum. Genet..

[32] Coutelier M., Coarelli G., Monin M.-L., Konop J., Davoine C.-S., Tesson C., Valter R., Anheim M., Behin A., Castelnovo G. (2017). A panel study on patients with dominant cerebellar ataxia highlights the frequency of channelopathies. Brain.

[33] Gauquelin L., Hartley T., Tarnopolsky M., Dyment D.A., Brais B., Geraghty M.T., Tétreault M., Ahmed S., Rojas S., Choquet K. (2020). Channelopathies are a frequent cause of genetic ataxias associated with cerebellar atrophy. Mov. Disord. Clin. Pr..

[34] Coutelier M., Blesneac I., Monteil A., Monin M.-L., Ando K., Mundwiller E., Brusco A., Le Ber I., Anheim M., Castrioto A. (2015). A recurrent mutation in CACNA1G alters Cav3.1 T-type calcium-channel conduction and causes autosomal-dominant cerebellar ataxia. Am. J. Hum. Genet..

[35] Lange L.M., Gonzalez-Latapi P., Rajalingam R., Tijssen M.A., Ebrahimi-Fakhari D., Gabbert C., Ganos C., Ghosh R., Kumar K.R., Lang A.E. (2022). Nomenclature of genetic movement disorders: Recommendations of the International Parkinson and Movement Disorder Society Task Force—An update. Mov. Disord..

[36] Renbaum P., Kellerman E., Jaron R., Geiger D., Segel R., Lee M., King M.C., Levy-Lahad E. (2009). Spinal muscular atrophy with pontocerebellar hypoplasia is caused by a mutation in the VRK1 gene. Am. J. Hum. Genet..

[37] Indelicato E., Boesch S. (2021). From genotype to phenotype: Expanding the clinical spectrum of CACNA1A variants in the era of next generation sequencing. Front. Neurol..

[38] Kordasiewicz H.B., Thompson R.M., Clark H.B., Gomez C.M. (2006). C-termini of P/Q-type Ca 2+ channel α1A subunits translocate to nuclei and promote polyglutamine-mediated toxicity. Hum. Mol. Genet..

[39] Gao H., Boustany R.-M.N., Espinola J.A., Cotman S.L., Srinidhi L., Antonellis K.A., Gillis T., Qin X., Liu S., Donahue L.R. (2002). Mutations in a novel CLN6-encoded transmembrane protein cause variant neuronal ceroid lipofuscinosis in man and mouse. Am. J. Hum. Genet..

[40] Tuermer A., Mausbach S., Kaade E., Damme M., Sylvester M., Gieselmann V., Thelen M. (2021). CLN6 deficiency causes selective changes in the lysosomal protein composition. Proteomics.

[41] Mole S.E., Williams R.E., Goebel H.H. (2005). Correlations between genotype, ultrastructural morphology and clinical phenotype in the neuronal ceroid lipofuscinoses. Neurogenetics.

[42] Faruq M., Narang A., Kumari R., Pandey R., Garg A., Behari M., Dash D., Srivastava A., Mukerji M. (2013). Novel mutations in typical and atypical genetic loci through exome sequencing in autosomal recessive cerebellar ataxia families. Clin. Genet..

[43] Rus C.-M., Weissensteiner T., Pereira C., Susnea I., Danquah B.D., Torres G.M., Rocha M.E., Cozma C., Saravanakumar D., Mannepalli S. (2022). Clinical and genetic characterization of a cohort of 97 CLN6 patients tested at a single center. Orphanet J. Rare Dis..

[44] Tripathy D., Vignoli B., Ramesh N., Polanco M.J., Coutelier M., Stephen C.D., Canossa M., Monin M.-L., Aeschlimann P., Turberville S. (2017). Mutations in TGM6 induce the unfolded protein response in SCA35. Hum. Mol. Genet..

[45] McMichael G., Bainbridge M.N., Haan E., Corbett M., Gardner A., Thompson S., van Bon B.W.M., van Eyk C.L., Broadbent J., Reynolds C. (2015). Whole-exome sequencing points to considerable genetic heterogeneity of cerebral palsy. Mol. Psychiatry.

[46] Tripathy R., Leca I., van Dijk T., Weiss J., van Bon B.W., Sergaki M.C., Gstrein T., Breuss M., Tian G., Bahi-Buisson N. (2018). Mutations in MAST1 cause mega-corpus-callosum syndrome with cerebellar hypoplasia and cortical malformations. Neuron.

[47] Ben-Mahmoud A., Al-Shamsi A.M., Ali B.R., Al-Gazali L. (2019). Evaluating the role of MAST1 as an intellectual disability disease gene: Identification of a novel *de novo* variant in a patient with developmental disabilities. J. Mol. Neurosci..

[48] Rodríguez-García M.E., Cotrina-Vinagre F.J., Gómez-Cano M.d.L., de Aragón A.M., Martín-Hernández E., Martínez-Azorín F. (2020). *MAST1* variant causes mega-corpus-callosum syndrome with cortical malformations but without cerebellar hypoplasia. Am. J. Med. Genet. Part A.

[49] Hecher L., Johannsen J., Bierhals T., Buhk J.-H., Hempel M., Denecke J. (2020). The clinical picture of a bilateral perisylvian syndrome as the initial symptom of mega-corpus-callosum syndrome due to a MAST1-gene mutation. Neuropediatrics.

[50] Sloboda N., Renard E., Lambert L., Bonnet C., Leheup B., Todosi C., Schmitt E., Feillet F., Feigerlova E., Piton A. (2023). MAST1-related mega-corpus-callosum syndrome with central hypogonadism. Eur. J. Med Genet..

[51] Gana S., Serpieri V., Valente E.M. (2022). Genotype–phenotype correlates in Joubert syndrome: A review. Am. J. Med. Genet. Part C Semin. Med Genet..

[52] Hong H., Joo K., Park S.M., Seo J., Kim M.H., Shin E., Cheong H.I., Lee J.H., Kim J. (2019). Extraciliary roles of the ciliopathy protein JBTS17 in mitosis and neurogenesis. Ann. Neurol..

[53] Chaki M., Airik R., Ghosh A.K., Giles R.H., Chen R., Slaats G.G., Wang H., Hurd T.W., Zhou W., Cluckey A. (2012). Exome capture reveals ZNF423 and CEP164 mutations, linking renal ciliopathies to DNA damage response signaling. Cell.

[54] Choi H.J.C., Lin J.-R., Vannier J.-B., Slaats G.G., Kile A.C., Paulsen R.D., Manning D.K., Beier D.R., Giles R.H., Boulton S.J. (2013). NEK8 links the ATR-regulated replication stress response and S phase CDK activity to renal ciliopathies. Mol. Cell.

[55] Miyake N., Fukai R., Ohba C., Chihara T., Miura M., Shimizu H., Kakita A., Imagawa E., Shiina M., Ogata K. (2016). Biallelic TBCD mutations cause early-onset neurodegenerative encephalopathy. Am. J. Hum. Genet..

[56] Ocampo-Chih C., Dennis H., Lall N., Pham N., Liang B., Verma S., Fresneda J.N. (2022). PEBAT, an intriguing neurodegenerative tubulinopathy caused by a novel homozygous variant in TBCD: A case series and literature Review. Pediatr. Neurol..

[57] Grønborg S., Risom L., Ek J., Larsen K.B., Scheie D., Petkov Y., Larsen V.A., Dunø M., Joensen F., Østergaard E. (2018). A Faroese founder variant in TBCD causes early onset, progressive encephalopathy with a homogenous clinical course. Eur. J. Hum. Genet..

[58] Verdura E., Rodríguez-Palmero A., Vélez-Santamaria V., Planas-Serra L., de la Calle I., Raspall-Chaure M., Roubertie A., Benkirane M., Saettini F., Pavinato L. (2021). Biallelic *PI4KA* variants cause a novel neurodevelopmental syndrome with hypomyelinating leukodystrophy. Brain.

[59] Salter C.G., Cai Y., Lo B., Helman G., Taylor H., McCartney A., Leslie J.S., Accogli A., Zara F., Traverso M. (2021). Biallelic PI4KA variants cause neurological, intestinal and immunological disease. Brain.

[60] Nothwang H.G., Kim H., Aoki J., Geisterfer M., Kübart S., Wegner R.D., van Moers A., Ashworth L.K., Haaf T., Bell J. (2001). Functional hemizygosity of PAFAH1B3 due to a PAFAH1B3-CLK2 fusion gene in a female with mental retardation, ataxia and atrophy of the brain. Hum. Mol. Genet..

[61] Johnson K.W., Smith K.A. (1991). Molecular cloning of a novel human cdc2/CDC28-like protein kinase. J. Biol. Chem..

[62] Kuczynska Z., Metin E., Liput M., Buzanska L. (2021). Covering the Role of PGC-1α in the Nervous System. Cells.

[63] Yang Y.N., Zhang M.Q., Yu F.L., Han B., Bao M.Y., Yan H., Li X., Zhang Y. (2023). Peroxisom proliferator-activated receptor-gamma coactivator-1alpha in neurodegenerative disorders: A promising therapeutic target. Biochem. Pharmacol..

[64] Wechsler D. (2014). Wechsler Intelligence Scale for Children.

[65] Sánchez-Monteagudo A., Álvarez-Sauco M., Sastre I., Martínez-Torres I., Lupo V., Berenguer M., Espinós C. (2020). Author response for “Genetics of Wilson disease and Wilson-like phenotype in a clinical series from eastern Spain”. Clin. Genet..

[66] Jumper J., Evans R., Pritzel A., Green T., Figurnov M., Ronneberger O., Tunyasuvunakool K., Bates R., Zidek A., Potapenko A. (2021). Highly accurate protein structure prediction with AlphaFold. Nature.

[67] Varadi M., Anyango S., Deshpande M., Nair S., Natassia C., Yordanova G., Yuan D., Stroe O., Wood G., Laydon A. (2022). AlphaFold Protein Structure Database: Massively expanding the structural coverage of protein-sequence space with high-accuracy models. Nucleic Acids Res..

[68] Schymkowitz J., Borg J., Stricher F., Nys R., Rousseau F., Serrano L. (2005). The FoldX web server: An online force field. Nucleic Acids Res..

[69] Venselaar H., Beek T.A.T., Kuipers R.K., Hekkelman M.L., Vriend G. (2010). Protein structure analysis of mutations causing inheritable diseases. An e-Science approach with life scientist friendly interfaces. BMC Bioinform..

