# Supplementary material for: Genetic Heterogeneity Underlying Phenotypes with Early-Onset Cerebellar Atrophy
Source: Int J Mol Sci. 2023 Nov 16;24(22):16400. doi: 10.3390/ijms242216400 (PMC10671053; doi:10.3390/ijms242216400)
Supplement: Supplementary file 1 [file ijms-24-16400-s001.zip › ijms-2638603-supplementary.pdf]

Supplementary Table S1. List of primers.

**Patient**	**Gene**	**Assay**	**Primer forward (5'-3')**	**Primer reverse (5'-3')**
MD-392	*CPLANE1*	Transcript analysis: splicing variant	TTGAACAAGGTGATGCTGGAC	GATGGAACTGCTAGATGATCG
		Transcript analysis: large deletion	AGAGAAATCCTGAGATGGTCC	ATTAGGCTCATGAAGTTGATCTG
MD-610	*PI4KA*	Transcript analysis: c.3845C>T; p.Ala1282_Asp1300del	CATTAGCAGTAAAGATTGTGACCC	GGACAGCTGAAGTAGTCAAAGG
MD-436	*CLK2*	Directed mutagenesis	AGTATAGGCTGCATCATCTTTGAACACTATGTGGGATTCACC	GGTGAATCCCACATAGTGTTCAAAGATGATGCAGCCTATACT
